# Microfluidic electro‐viscoelastic manipulation of extracellular vesicles

**DOI:** 10.1002/2211-5463.70080

**Published:** 2025-07-09

**Authors:** Seyedamirhosein Abdorahimzadeh, Éva Bozó, Zikrullah Bölükkaya, Artem Zhyvolozhnyi, Anatoliy Samoylenko, Henrikki Liimatainen, Seppo J. Vainio, Caglar Elbuken

**Affiliations:** ^1^ Disease Networks Research Unit, Faculty of Biochemistry and Molecular Medicine University of Oulu Finland; ^2^ Kvantum Institute University of Oulu Finland; ^3^ Fiber and Particle Engineering Research Unit, Faculty of Technology University of Oulu Finland; ^4^ Biomedicine Research Unit, Faculty of Medicine University of Oulu Finland; ^5^ VTT Technical Research Centre of Finland Ltd. Oulu Finland

**Keywords:** electrokinetics, extracellular vesicles, microfabrication, microfluidics, viscoelasticity

## Abstract

Microfluidic technology has created new opportunities for developing innovative tools for biological applications. Given the significance of extracellular vesicles (EVs), extensive research has focused on developing microfluidic techniques for EV isolation. This research protocol presents electro‐viscoelastic microfluidics as a novel approach for manipulating EVs. The system leverages the viscoelasticity of the suspending medium along with an externally applied electric field to alter EV motion within a microchannel. These findings suggest that our electro‐viscoelastic microfluidic system has the potential for further development to be used for EV isolation.

## Graphical abstract

The electro‐viscoelastic manipulation as a potential method for separation of particles based on size. The particles introduced as a sheath flow migrate to the channel center under the influence of simultaneously applied electric field and pressure driven flow.

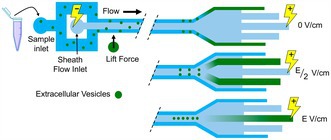

**Abbreviations**
CAD: computer‐aided design
DMEM: Dulbecco's modified eagle medium
EDTA: ethylenediaminetetraacetic acid
EGFP: enhanced green fluorescent protein
EV: extracellular vesicles
FBS: fetal bovine serum
IPA: isopropyl alcohol
MW: molecular weight
PBS: phosphate‐buffered saline
PDMS: polydimethylsiloxane
PEO: polyethylene oxide

Extracellular vesicles (EVs) are lipid bilayer‐bound entities released by cells into their surrounding medium [1, 2]. EVs carry biologically significant macromolecules, such as proteins, DNA, and RNA [3, 4]. Their ubiquitous presence in biofluids, such as blood and urine, along with their biologically important cargo, has made them a valuable target for minimally invasive disease screening, including cancer detection [5, 6]. Furthermore, given their role in intercellular communication and their ability to pass through biological barriers, EVs are being explored for therapeutic applications [7].

An indispensable aspect of EV research is their isolation from the carrying medium. However, the high heterogeneity of EVs and the presence of other components with similar physical and chemical properties make obtaining a pure EV sample a challenging and labor‐intensive task [8]. Ultracentrifugation is considered the gold standard for EV isolation [9], although it typically results in low EV recovery and coprecipitates nonvesicular components with similar physical properties. Additionally, ultracentrifugation is time‐consuming and costly due to its reliance on specialized equipment [10]. Other bulk methods, such as size‐exclusion chromatography, ultrafiltration, and polymer‐based precipitation, have been explored to improve EV isolation. Yet, no single method fully addresses all the challenges associated with EV isolation [11]. Therefore, novel approaches must be explored to enable more efficient and convenient EV isolation.

Microfluidics has shown great potential for the controlled manipulation and isolation of biological matter [12]. Specifically, various microfluidic techniques have been developed for cell isolation, among which inertial microfluidics has demonstrated the capacity for clinically relevant high‐throughput cell isolation [13]. Efforts have also been made to isolate EVs using microfluidic technology [14]. Viscoelastic microfluidics, which utilizes a viscoelastic medium as the carrier fluid in microchannels, has emerged as a promising technique for EV isolation [15]. For example, Meng et al. introduced a viscoelastic microfluidic platform capable of isolating EVs from blood. Their system comprised two sequential isolation modules: the first removed large components, such as cells, while the second fractionated EVs based on size [16].

Inspired by our previous studies on particle electrohydrodynamics in microchannels [17, 18, 19], here, we aim to further advance viscoelastic microfluidics by integrating an external electric field, introducing electro‐viscoelastic microfluidics. The use of the electric field increases the net force exerted on particles by orders of magnitude, which is crucial for manipulating nanoparticles. Furthermore, incorporating an external electric field adds another degree of control over particle behavior, as the applied force can be adjusted by modifying the electric field's magnitude.

Figure 1 presents a schematic of the electro‐viscoelastic system. As shown in the figure, the EV sample (spiked into polyethylene oxide polymer solution) is positioned near the channel walls with the aid of another viscoelastic solution (250 p.p.m. PEO) serving as the sheath flow. The application of an external electric field induces the lateral migration of EVs toward the channel center, with the extent of migration depending on the field's magnitude.

**Fig. 1:**
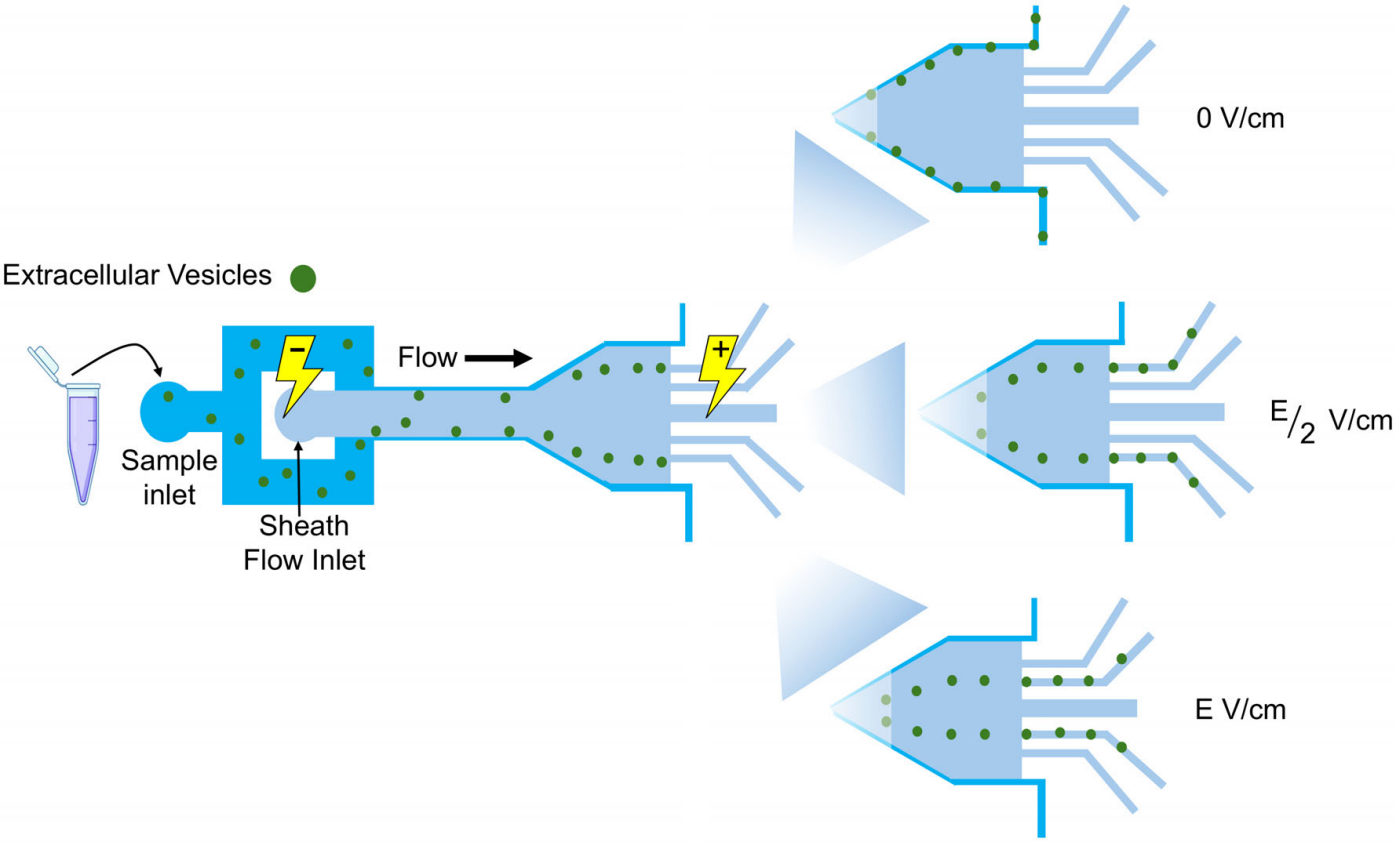
Lateral migration of extracellular vesicles in an electro‐viscoelastic microfluidic system. Under an external electric field, extracellular vesicles (EVs) suspended in a viscoelastic solution migrate toward the channel center, with the extent of migration depending on the field strength.

In the following sections, we provide a detailed research protocol to set up an electro‐viscoelastic system and we demonstrate its performance in manipulating cancer cell‐derived EVs. Electro‐viscoelastic microfluidics has the potential to become the next‐generation microfluidic platform for efficiently separating small‐sized particles, including EVs.

## Materials

### Preparation of the viscoelastic solution

The viscoelastic solution was prepared by dissolving polyethylene oxide (PEO; Sigma‐Aldrich, St. Louis, MO, USA) in deionized water. To obtain a 250 p.p.m. PEO solution, 0.25 g of PEO powder (MW = 900 000 Da; Sigma‐Aldrich) was measured using a precision balance. The PEO powder was then slowly added to 500 mL of deionized water to prevent clumping. After mixing, additional deionized water was added until the total volume reached 1 L. The solution was stirred overnight using a magnetic stirrer.

### Fabrication of the microfluidic chip

**Fig. 2:**
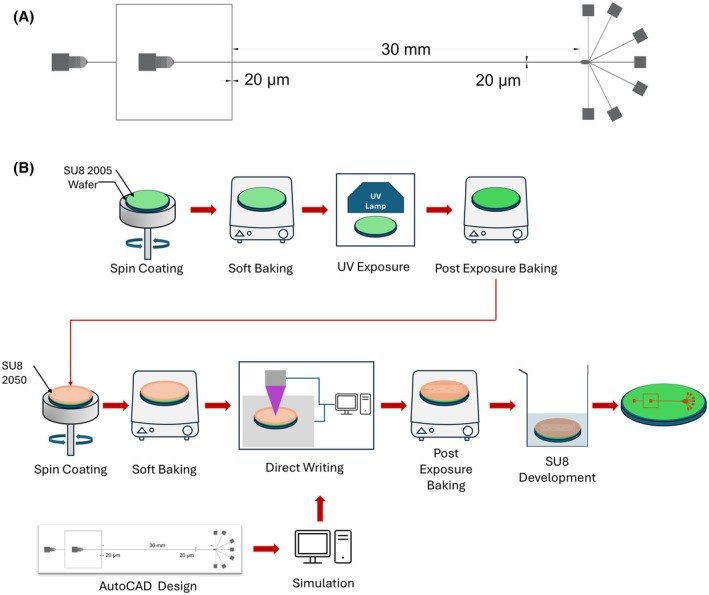
(A) Computer‐aided design (CAD) design of the microfluidic system. (B) Fabrication protocol of the microfluidic system.

The standard soft lithography process was used to fabricate the microfluidic chip (with a uniform height of 50 μm) via the following steps (Fig. 2):

1. Preparation of the 2D CAD design

The CAD design of the microfluidic channel was created using AutoCAD. The channel width and main channel length were set to 20 μm and 30 mm, respectively (Fig. 2A).

2 Fabrication of the silicon mold in the cleanroom
  The following steps are carried out in the cleanroom to create a silicon mold with microfluidic structures patterned on its surface at a height of 50 μm (Fig. 2B).

- First, a base layer of SU‐8 is coated on a silicon wafer to prevent PDMS adhesion during replica molding. The first 5 μm layer is formed using SU‐82005 (Kayaku Advanced Materials, Westborough, MA, USA).
- Pour 2 mL of SU‐82005 and distribute over a 4‐inch single‐sided polished wafer (University Wafer, South Boston, MA, USA). Wafers with a thickness of 500 μm (or larger) give structural support and enable ease‐of‐handling.
- Spin coating: spin‐coat the wafer using the following parameters (Table 1).
- Soft baking: place the SU‐8 coated silicon wafer on two hot plates (Thermo Fisher Scientific, Waltham, MA, USA) set at 65 and 95 °C to allow a ramped heating (Table 2). Ensure uniform heating of the wafer to avoid cracks in the SU‐8 layer.
- UV exposure: the first layer of SU‐8 is exposed to UV light (365 nm) with a total exposure energy of 120 mJ·cm^−2^ without using any mask (Karl Suss MA‐6 contact aligner).
- Postexposure baking: place the exposed wafer on the hot plates in the following order (Table 3).
- The next step involves coating a layer of SU‐82050, which generates the final pattern of the microfluidic structures on the silicon wafer and sets the channel height to 50 μm.
- Pour SU‐82050 (Kayaku Advanced Materials) on the wafer center to cover approximately half of the wafer area.
- Spin coating: spin‐coat the wafer using the following parameters (Table 4).
- Soft baking: place the wafer on the hot plates in the following order (Table 5).
- Direct writing of the microfluidic patterns: we used a direct writer system (Kloé Dilase 125; KLOÉ, Saint‑Mathieu‑de‑Tréviers, France) to generate the microfluidic patterns on the SU‐8 coated wafer. Alternatively, you can use UV exposure with a mask.
- Place the wafer into the direct writer system. Make sure the wafer is positioned properly on the stage and the vacuum is on before starting the writing process.
- Upload the writing file (.LWO) generated using the direct writer software (Kloé Design) and start the writing process using the following parameters (Table 6).
- Postexposure baking: place the exposed wafer on the hot plates in the following order (Table 7).
- Development: after the postexposure baking, wait for a few minutes to allow the wafer to cool to room temperature. Then, place the wafer in the developer (Kayaku Advanced Materials) to remove the uncured SU‐8 for around 10 min with gentle shaking.
- The mold is then ready to be cast with PDMS (polydimethylsiloxane).

3 Casting PDMS and making the final PDMS chips.

- Mix PDMS base and the curing agent in a 10 : 1 ratio (SYLGARD 184 Silicone Elastomer Kit; Dow, Midland, MI, USA).
- Place the mixture under vacuum to remove air bubbles.
- Pour the degassed PDMS onto the silicon mold, which is placed inside an aluminum weighing dish (Fisher Scientific, Pittsburgh, PA, USA).
- Cure the PDMS in the oven at 80 °C for 120 min.
- After the mold cools down to room temperature, peel off the PDMS from the mold and cut the chips to the size of a glass microscope slide (Superfrost™Plus Adhesion Microscope Slides; Epredia, Portsmouth, NH, USA),
- Place the glass slide and PDMS channel in an oxygen plasma chamber (Gambetti Kenologia Srl, Milan, Italy). Set the parameters at 100 W for 25 s.
- After plasma treatment, place the exposed surface of the glass slide on top of the PDMS channel and apply gentle pressure to ensure bonding.
- Place the chip on the hot plate (Thermo Fisher Scientific) at 80 °C for an hour to further strengthen the bonding.
- The microfluidic chip is ready for use.

**Table 1:** Spin coating parameters for the first layer of SU8 coating (2005).

	Time	Ramp	Speed
First step	25 s	100 rpm·s^−1^	500 rpm
Second step	40 s	200 rpm·s^−1^	2500 rpm

**Table 2:** Soft baking temperatures and durations for the first layer of SU8 coating (2005).

65 °C	95 °C	65 °C
2 min	4 min	1 min

**Table 3:** Postexposure baking temperatures and durations for the first layer of SU8 coating (2005).

65 °C	95 °C	65 °C
1 min	3 min	1 min

**Table 4:** Spin coating parameters for the second layer of SU8 coating (2050).

	Time	Ramp	Speed
First step	55 s	100 rpm·s^−1^	500 rpm
Second step	40 s	300 rpm·s^−1^	3500 rpm

**Table 5:** Soft baking temperatures and durations for the second layer of SU8 coating (2050).

65 °C	95 °C	65 °C
2 min	7 min	1 min

**Table 6:** Laser writing parameters used for patterning microstructures on the SU‐82050‐coated layer.

Modulation	Writing speed	Replacement speed	UV focal plane height	Laser line separation distance
40 (%)	50 (mm·s^−1^)	1–3 (mm·s^−1^)	−0.83 (μm)	5 (μm)

**Table 7:** Postexposure baking temperatures and durations for the second layer of SU8 coating (2050).

65 °C	95 °C	65 °C
3 min	7 min	1 min

### EV sample preparation

We isolated EVs from a genetically modified 786‐O cell line that stably expresses a membrane‐targeted form of EGFP. EVs produced by these cells carry palmitoylated EGFP protein and are therefore characterized by green fluorescence [20] which allows EV tracking. Further details can be found in our previous study [21]. The protocol used to obtain the EV samples for our experiments is outlined below.

#### Cell culture

- Defreeze the human renal adenocarcinoma 786‐O cells (300 107; CLS Cell Lines Service, Eppelheim, Germany) that stably overexpress membrane‐bound (palmitoylated) form of EGFP [20].
- Culture the cells in Dulbecco's Modified Eagle Medium (DMEM)/F‐12 (31 331 028; Gibco, Thermo Fisher Scientific) supplemented with:
  ○ 10% fetal bovine serum (FBS) (F7524‐500 mL; Sigma‐Aldrich)
  ○ 100 U·mL^−1^ penicillin and 100 μg·mL^−1^ streptomycin (P0781‐100 mL; Sigma‐Aldrich).
- Culture conditions: 37 °C in 5% CO_2_.

1. Cell passaging

- Wash cells with 1× PBS (20‐031‐CV; Corning, Corning, NY, USA) and split using trypsin–EDTA solution (25 200–072; Gibco).
- Check cell viability using Trypan Blue Stain (T10282; Invitrogen, Carlsbad, CA, USA) and the TC20 Automated Cell Counter (145–0101; Bio‐Rad, Hercules, CA, USA). Cell viability should be within the range of 90–97%.
- Cultivate cells until they reach approximately 80% confluence.

1. Experimental setup

- Plate 5 × 10^6^ cells per 15 cm dish (639 160; Greiner Cellstar, Monroe, NC, USA) (15 dishes total).
- Allow cells to grow in 15‐cm dishes until reaching 80% confluence.
- Replace the medium with FBS‐free medium (20 mL per dish) and incubate cells for 24 h.

1. Medium collection and processing

- Collect 300 mL of conditioned medium.
- Centrifuge the collected medium at 5000 ***g*** for 15 min to remove floating cells and debris.
- Transfer the supernatant to fresh tubes.
- Concentrate supernatant using Centricon Plus‐70 filter units (cut‐off of 100 kDa; Merck Millipore, Burlington, MA, USA) according to the manufacture's protocol.
- Store the concentrated EV‐enriched sample at −70 °C.

1. Final experimental sample preparation

- Defreeze the concentrated EV‐enriched sample.
- Exchange the suspending medium of the EV‐enriched sample with Milli‐Q water using a 12–14 000 Da cellulose membrane (Medicell visking dialysis tubing) for 2 h to reduce conductivity (original conductivity: 14.40 mS·cm^−1^).
- Suspend the resulting sample after buffer exchange in 500 p.p.m. PEO at a 1 : 1 ratio, resulting in a low conductivity (< 200 μS·cm^−1^).

### Experimental setup

**Fig. 3:**
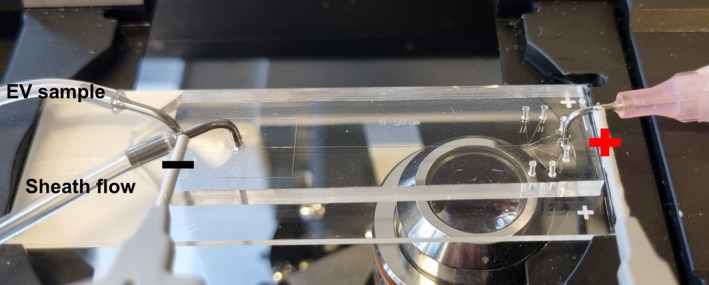
E‐viscoelastic microfluidic system with its electrical and fluidic connections.

An inverted microscope (Zeiss Axio Observer; Zeiss, Oberkochen, Baden‑Württemberg, Germany) was utilized for visualization. The sample and sheath solutions were introduced into the microchip via a pressure pump (Elveflow OB1; Elveflow, Paris, Île‑de‑France, France). To generate the externally applied electric field, a high‐voltage power supply (HVS448 3000 V; LabSmith, Livermore, CA, USA) was connected to the dispensing tips at the sheath inlet and the middle outlet using micro clips (Fig. 3). A monochrome camera (Axiocam 807 mono; Zeiss) was used to record the trajectories of fluorescent particles (EVs) in the expansion region. The acquired images were processed with the Zeiss software (zen 3.9) and further analyzed in matlab and originpro to visualize the particle distribution.

## Methods

Figure 3 shows the microfluidic system with its fluidic and electrical connections.

1. Place the microfluidic chip on the microscope stage.
2. Connect a 250 p.p.m. PEO solution to the sheath flow inlet. Use a 20 gauge, 90‐degree angled syringe metal tip for connection (METCAL; Cypress, CA, USA).
  Before connecting the metal tip to the sheath flow inlet, apply a low pressure (e.g., 50 mbar) to fill the tubing with the solution and ensure that liquid is dripping from the metal tip. Then, connect it to the sheath flow inlet. This step prevents air bubbles from entering the microchannel. Gradually increase the pressure to 1500 mbar and be mindful of any leaks due to faulty bonding.
3. Check all channels under the microscope to ensure no air bubbles are trapped.
  The expansion region and the outlet channels are particularly prone to air bubbles. Try to eliminate them by increasing the pressure or pushing them out by gently pressing on the PDMS over the channels.
4. Connect the EV sample to the sample inlet.
  Follow the same procedure described in Step 2. After connecting the EV sample, increase the pressure to match that of the sheath flow to prevent backflow of the sheath flow into the EV sample. This pressure setting (sample and sheath flow inlets both pressurized at 1500 mbar) results in flow rates of around 2 and 5 μL·min^−1^ for the sample and sheath flow, respectively. These values have been obtained by calculating the hydraulic resistances of the microchannels $\left(\text{flow rate}=\frac{\text{Pressure}}{\text{Hydraulic resistance}}\right)$ and validated by experiment. You may also explore other pressure settings.
5. Check the intersection where the EV sample and the sheath flow meet under the microscope.
  Ensure that the fluorescent signal of the EVs is visible and that the sheath flow effectively pushes them toward the sidewalls.
6. Insert a metal tip in the middle outlet, which will serve as the positive electrode.
7. Connect the high‐voltage power supply to the microfluidic chip.
  Connect the negative pole to the metal tip carrying the sheath flow and the positive pole to the metal tip inserted in the middle outlet. Ensure that there are no water leaks or unintended electrical pathways connecting the electrodes to the microscope stage to avoid short circuits.
8. Apply the electric field by increasing the voltage and observe the lateral migration of EVs in response to changes in the applied electric field.
  As the electric field is applied, EVs begin migrating toward the channel center. Increasing the electric field strength enhances the center‐focusing force on EVs, further pushing them toward the middle of the channel.

### Tips and tricks

1. In case of channel clogging, place the chip in a sonicator bath and flush the channel with deionized water or ethanol/IPA using a syringe. Sonication significantly improves the cleaning process.
2. It is highly recommended that this washing procedure be performed after each experiment, even if there is no visible clogging or debris inside the channel. If not cleaned, any remaining viscoelastic solution in the channel will dry over time and leave polymer residue.
3. While connecting or disconnecting the tubings from the chip or making any physical changes to the system, make sure that the power supply is disconnected.
4. The behavior of the polymer solution can vary over time. It is specifically recommended not to use the solution immediately after preparation. Based on experience, the solution becomes stable a few days after preparation. Additionally, it is advised to renew the polymer solution after 1 month. Rheological characteristics of the solution can be used for standardization.

## Discussion

**Fig. 4:**
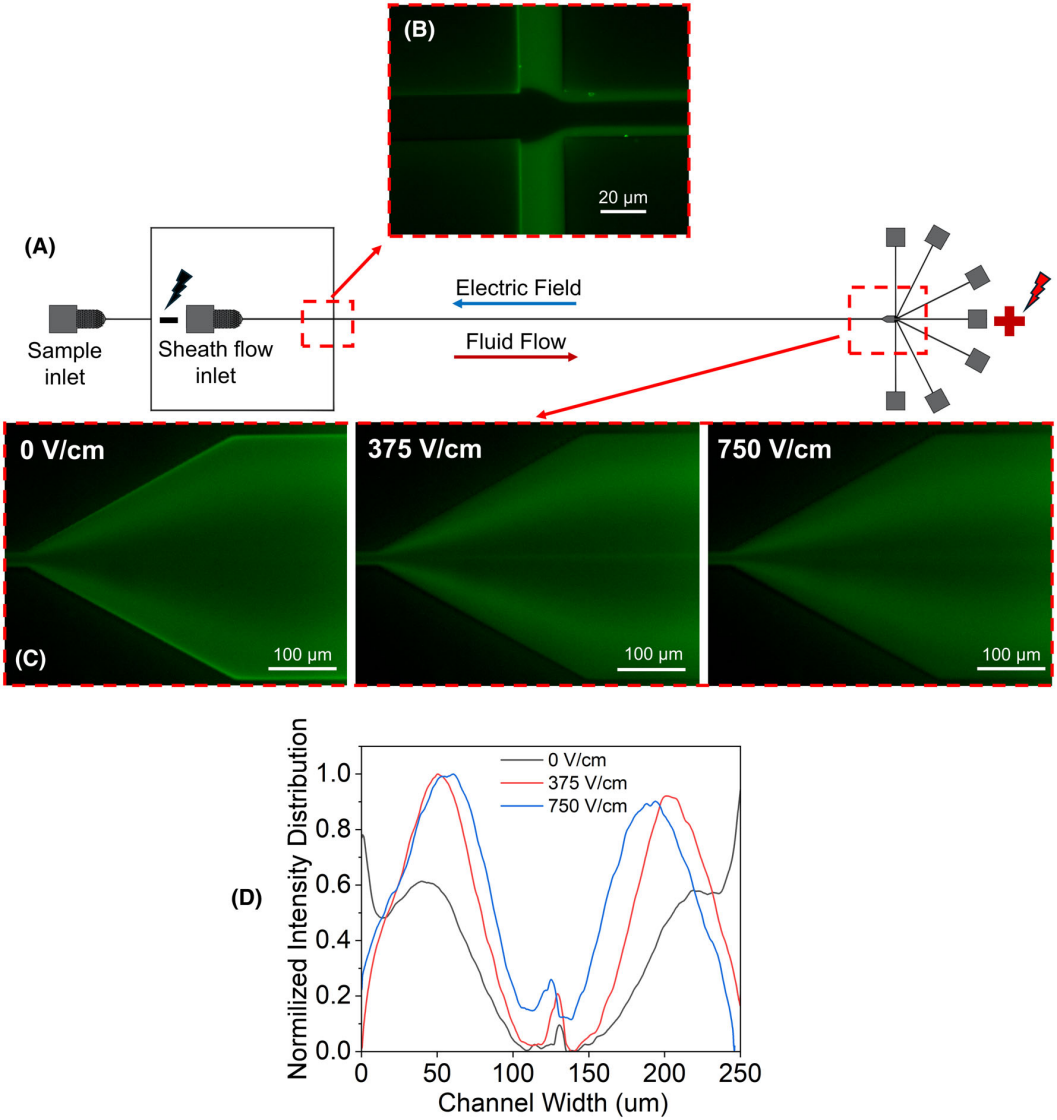
Lateral migration of extracellular vesicles (EVs) under the effect of electro‐viscoelastic force. (A) Schematic of the microfluidic chip. (B) At the intersection of the sheath flow and the sample flow, EVs are pushed toward the top and bottom walls. (C) Upon applying an external electric field, EVs begin migrating toward the channel center, with the extent of migration depending on the field strength. (D) Normalized fluorescent intensity signal distribution for the three cases of 0, 375, and 750 V·cm^−1^ electric fields shown in (C).

Figure 4 shows the evolution of EVs lateral migration in the channel upon application of the external electric field. Due to the negative charge of the EVs—with a zeta potential of approximately −20 mV for renal adenocarcinoma cell‐derived EVs [22]—they are expected to experience an electrophoretic velocity along the same direction as the main fluid flow. This additional velocity results in EVs leading the background fluid. Previous theoretical and experimental studies have reported that in electro‐viscoelastic systems particles experience a lift toward the center of the channel in this condition [23, 24]. Notably, this behavior is opposite to particle behavior in Newtonian fluids under the same condition, which is explained by the Saffman force [25, 26]. The extent of lateral migration depends on the magnitude of the electric field. However, the change between 0 and 375 V·cm^−1^ is more significant than between 375 and 750 V·cm^−1^. This observation can be explained by two factors.

On the one hand, the electro‐viscoelastic force is dependent on shear, which is maximized near the channel boundaries. Therefore, particles experience a stronger force in these regions. On the other hand, as particles move closer to the channel center, they travel faster along the channel length due to the parabolic velocity profile. Consequently, their residence time in the channel decreases, leading to reduced lateral displacement under the electro‐viscoelastic force.

Electro‐viscoelastic microfluidics paves the way for efficient manipulation and separation of small particles by enhancing the lateral force acting on them and providing an additional degree of control over particle dynamics in a microchannel. Electro‐viscoelastic microfluidic platform can be used to fractionate particle mixtures containing particles of various sizes and charges. Electro‐viscoelastic microfluidics can enhance the resolution of size‐based particle separation and, more importantly, establish itself as a novel charge‐based separation technique, which would hold a significant place in bioanalytical research.

## Conflict of interest

The authors declare no conflict of interest.

## Author contributions

SA contributed to the conceptualization, data curation, formal analysis, methodology, and writing—original draft. ÉB and ZB contributed to the data curation, investigation, and writing—original draft. AZ contributed to the resources, investigation, and writing—original draft. AS contributed to the resources, supervision, and writing—review and editing. HL contributed to the funding acquisition, project administration, supervision, and writing—review and editing. SJV contributed to the funding acquisition, project administration, supervision, and writing—review and editing. CE contributed to the conceptualization, funding acquisition, methodology, project administration, resources, supervision, and writing—review and editing.

## Data Availability

The data that support the findings of this study are available from the corresponding author upon reasonable request.
